# Clinical Application of an Augmented Reality Navigation System for Transforaminal Epidural Injection: A Randomized Controlled Trial

**DOI:** 10.3390/jcm13071992

**Published:** 2024-03-29

**Authors:** Yookyung Jang, Sunghwan Lim, Sunhee Lee, Lee Gyeong Je, Taesan Kim, Subin Joo, Joonho Seo, Deukhee Lee, Jae Chul Koh

**Affiliations:** 1 Department of Anesthesiology and Pain Medicine, Korea University College of Medicine, Seoul 02841, Republic of Korea; gamtang@korea.ac.kr (Y.J.); sunfanooo@gmail.com (S.L.); jellyg0209@gmail.com (L.G.J.); taesan9494@kumc.or.kr (T.K.); 2 Center for Healthcare Robotics, Artificial Intelligence and Robotics Institute, Korea Institute of Science and Technology, Seoul 02792, Republic of Korea; slim@kist.re.kr (S.L.); dkylee@kist.re.kr (D.L.); 3 Department of Medical Assistant Robot, Korea Institute of Machinery and Materials, Daegu 42994, Republic of Korea; subin@kimm.re.kr (S.J.); jhseo@kimm.re.kr (J.S.)

**Keywords:** augmented reality, navigation, transforaminal epidural injection, pain management

## Abstract

**Objectives:** Augmented reality (AR) navigation systems are emerging to simplify and enhance the precision of medical procedures. Lumbosacral transforaminal epidural injection is a commonly performed procedure for the treatment and diagnosis of radiculopathy. Accurate needle placement while avoiding critical structures remains a challenge. For this purpose, we conducted a randomized controlled trial for our augmented reality navigation system. **Methods:** This randomized controlled study involved 28 patients, split between a traditional C-arm guided group (control) and an AR navigation guided group (AR-NAVI), to compare procedure efficiency and radiation exposure. The AR-NAVI group used a real-time tracking system displaying spinal structure and needle position on an AR head-mounted display. The procedural time and C-arm usage (radiation exposure) were measured. **Results:** All patients underwent successful procedures without complications. The AR-NAVI group demonstrated significantly reduced times and C-arm usage for needle entry to the target point (58.57 ± 33.31 vs. 124.91 ± 41.14, *p* < 0.001 and 3.79 ± 1.97 vs. 8.86 ± 3.94, *p* < 0.001). **Conclusions:** The use of the AR navigation system significantly improved procedure efficiency and safety by reducing time and radiation exposure, suggesting a promising direction for future enhancements and validation.

## 1. Introduction

When performing medical procedures, it is crucial to locate the appropriate tools to the correct target. Moreover, establishing the correct insertion point and target orientation is essential. Various imaging modalities, such as X-rays and ultrasound, have been employed for guidance in these procedures [1,2]. However, even when guided by these imaging modalities, cognitively reconstructing the actual three-dimensional structure and determining the orientation of instruments remains a challenge due to their provision of two-dimensional information. Although navigation systems have been developed to overcome these problems [3,4,5], they are often unintuitive and inconvenient. Those monitor-based navigation systems commonly accompany clinicians’ attention shift problems and hand–eye coordination problems. It is often difficult to manipulate the tool while looking at the remote monitor displaying the navigational information. During surgical procedures, distractions could pose a risk by shifting the clinician’s attention, potentially impairing surgical outcomes [6]. To address this attention shift and eye–hand discrepancy problems, a guiding system using augmented reality has been developed and applied in surgical procedures [7,8,9,10]. However, most of these systems are only applicable to large, rigid instruments for patients who are immobilized under general anesthesia. In addition, they face challenges in integrating them into outpatient treatment because they require markers to be invasively fixed to the patient or require heavy and large equipment [11].

Lower back pain with radicular symptoms represents a prevalent cause of disability among the adult population [12,13,14]. Lumbosacral transforaminal epidural injection is a commonly performed procedure for the treatment and diagnosis of radiculopathy [15]. Although this procedure is relatively safe and uncomplicated, accurately positioning the needle to avoid bones and nerves can be challenging in patients with severe anatomical degeneration or deformities, potentially increasing the risk of complications [16]. In addition, the current C-arm-guided interventions are associated with increased radiation exposure for both clinicians and patients [17]. Notably, the level of radiation exposure escalates in scenarios involving operators with lesser skill or in patients presenting anatomical variations that complicate the procedure. In response, we developed an augmented reality navigation system that can be easily applied in the outpatient setting. The feasibility of this system was successfully verified in our previous studies using simulation phantoms [18,19]. However, to the best of our knowledge, no reports have been published on the clinical application of such an augmented-reality-guided system in an outpatient setting.

We planned this study based on the experimental results obtained from models and animal studies, which lead us to believe in the feasibility of the system’s clinical application. Therefore, we conducted this AR-guided navigation system’s randomized controlled trial in real patients for the first time.

## 2. Materials and Methods

This randomized controlled trial received approval from the institutional review board (IRB) of the authors’ affiliated institutions (Korea University Anam Hospital, Republic of Korea; approval number, 2022AN0504; date of approval, 3 November 2022) and the Ministry of Food and Drug Safety of Korea (approval number, 1422; date of approval, 3 November 2022). The study ran from 29 March 2023 to 22 August 2023. The study was registered at the Clinical Research Information Service Protocol Registration System (ref: CRIS, KCT0009265). The development of the software and system used in this study involved collaboration with multiple organizations, including the Korea University College of Medicine (Seoul, Republic of Korea), Korea Institute of Science and Technology (Seoul, Republic of Korea), Korea Institute of Machinery and Materials (Seoul, Republic of Korea), as well as MetaSystems Co., Ltd. (Seongnam-si, Republic of Korea), and Digitek Co., Ltd. (Seoul, Republic of Korea). The project spanned a duration of 5 years and was conducted within the framework of the Technology Development Program for the Convergence of AI, Bio, Robotics, and Medicine.

### 2.1. Preparation of AR-Guided Navigation System

We used an AR-assisted navigation system developed by our team. The feasibility of this system was ascertained through the utilization of simulation phantoms in our previous studies [18,19]. This system comprises several key components: planning and navigation software based on virtual reality, an advanced system for tracking both the patient and surgical tools [20], and an AR-assisted navigation module that seamlessly integrates with a head-mounted display (HMD, HoloLens 2, Microsoft, Redmond, WA, USA). In addition, we revisited and revamped the graphical user interface of the navigation software to align it with the trial requirements. Importantly, all of these modifications were subjected to discussions and consultations with physicians to ensure their congruence with the clinical needs.

### 2.2. Study Population

The study involved patients aged 20 years or older, presenting with neurological symptoms including back pain rated at 3 or higher on the pain scale or unilateral lower extremity pain without signs of paralysis or sensory loss; lumbar disc herniation or degenerative disease with symptoms consistent with the neuromuscular distribution stimulated by the lesion observed on computed tomography (CT) or magnetic resonance imaging; and a positive result in the straight-leg elevation test during the physical examination. Only patients who voluntarily consented to participate in the clinical trial and provided written consent were included in the study. Exclusion criteria comprised patients who had a blood coagulation disorder (international normalized ratio >1.5 or platelet count <100,000/mm^2^) or who were taking anticoagulants; developed an infection in the systemic or injection site; were allergic to certain medications (contrast media, local anesthetics, and steroids); were allergic to skin markers; were pregnant or lactating; were unable to assume a prone position; were indicated for emergent surgery due to the presence of a neurologic injury; and who exhibited cognitive decline.

### 2.3. Evaluation

The participants were randomly assigned to the AR-NAVI and control groups in a 1:1 ratio. The group allocation was carried out according to a pre-generated randomization table and the order of assigned patient number. Block randomization was used to ensure a balanced distribution between the groups. The randomization code was generated using the R Statistical Software (version 4.0.3; R Core Team, R Foundation for statistical Computing, Vienna, Austria). This task was carried out by a clinical research coordinator (CRC) who was not involved in the patient assessment or the procedures. The group allocation was subsequently concealed in a sealed and opaque envelope by the same CRC. Upon a participant’s enrollment in the study, the physician would be informed of the group assignment. While the physician was aware of the patient’s allocation to the AR-NAVI group, both the patients and the assessors responsible for evaluation were kept unaware of the group allocation.

**Figure 1:**
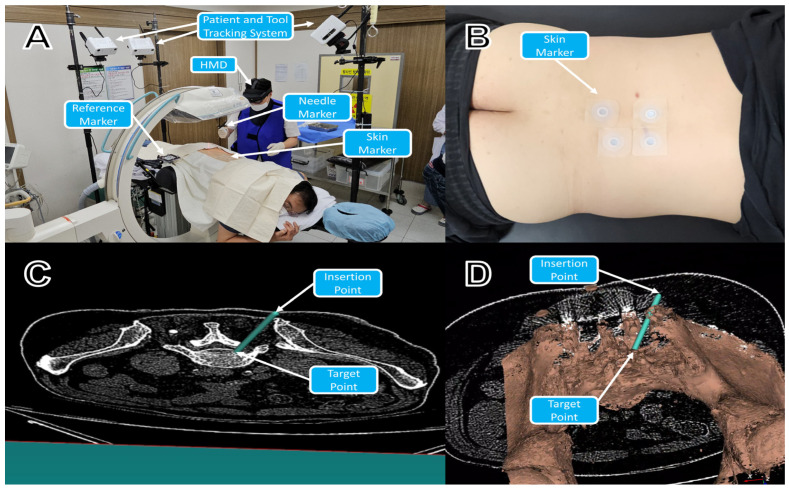
Preparation before the procedure in AR-NAVI group. (**A**) An augmented-reality (AR)-assisted navigation system (three optical trackers, navigation software, a wireless router, and a reference marker) were installed in the procedure room. (**B**) Four skin markers were attached to the dorsal surfaces of the patients. (**C**) The target and insertion points were established on the resliced images of the CT volume for the procedure. (**D**) A 3D model of the spinal bone was generated using an isosurface extraction approach.

Before performing the procedure, an AR-assisted navigation system (comprising three optical trackers, a personal computer [PC] with navigation software, a wireless router, and a reference marker) was installed in the procedure room (Figure 1A). Four skin-adhesive markers were affixed to the dorsal surfaces of both patient groups (Figure 1B). CT scans were performed with patients in the prone position with dorsal surface markers attached. After acquiring the image through a CT scan, each participant laid in a prone position on a table situated in the procedure room. The Digital Imaging and Communications in Medicine (DICOM) files of patients in the AR-NAVI group were used to generate three-dimensional (3D) volume data. From the volume data, the 3D polygon models of skin-attached markers, dorsal surface of the skin, and lumbar spines were created using the isosurface extraction approach [21]. Prior to the procedure, the target and insertion points were determined on the resliced image of the CT volume (Figure 1C) while visualizing a 3D polygon model of the lumbar spine (Figure 1D). After selection of the target and insertion points, the 3D polygon model of the lumbar spine, target, and insertion point data were transmitted to an HMD (HoloLens 2, Microsoft, Redmond, WA, USA) through a wired connection.

**Figure 2:**
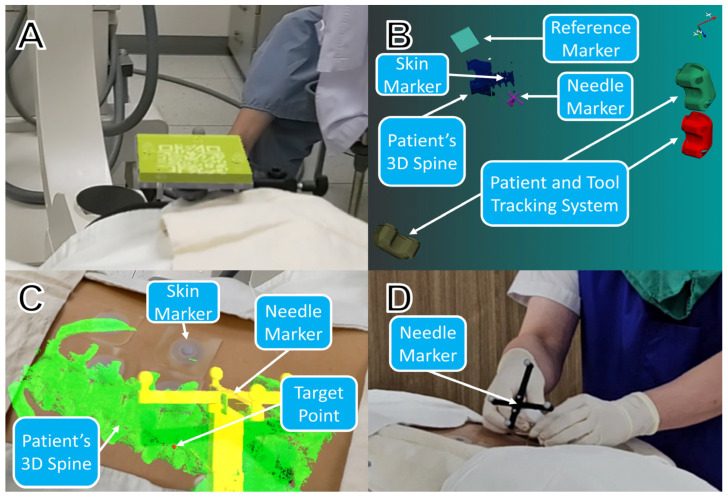
An AR-guided navigation system was employed during the procedure. (**A**) The 3D model of the reference marker in the AR view is aligned with the reference marker in the real view. (**B**) Relative spatial coordinates of the skin, needle markers, and patient’s 3D spine model were provided based on the coordinates of the reference marker in real time. (**C**) Visualization of the patient’s 3D spine model, target point, and tracked needle model on the head-mounted display (HMD). (**D**) Needle insertion with an easily detectable marker during the procedure.

In the AR-NAVI group, the AR-assisted navigation software was executed on the HMD and PC in the procedure room. The created 3D models were loaded, and the connection between the tracker and HMD was confirmed. After wearing the HMD, the physician assessed whether the 3D model of the reference marker in the AR view was properly aligned with the reference marker in the real world (Figure 2A). The positions of the skin and needle markers were determined in real time using three trackers installed in the procedure room, and the tracking data were utilized to calculate the relative spatial coordinates based on the coordinate of the reference marker. The relative spatial coordinates were then transmitted to the navigation software through a wireless network (Figure 2B). By transmitting this tracking information to the HMD through a wireless network, the procedure could be performed by intuitively recognizing the spatial location of the needle and the patient’s spinal structure. The physician wearing the HMD could visualize or hide the virtual 3D models projected on the patient’s body, such as the spinal structure, target point, and insertion point (Figure 2C). For this procedure, a rigid-body marker attached to the epidural needle was redesigned to improve the stability of the needle pose tracking. The needle markers included four spherical retro-reflective markers. The needle marker was developed to allow easy engagement and detachment of the needles as needed (Figure 2D).

In the procedure room, patients in both groups are positioned prone on the table. A 22-gauge needle for spinal anesthesia (UNISIS Corp., Tokyo, Japan) was employed in both groups. All procedures were performed by a physician who wore an HMD in both groups. For the control group, the C-arm is utilized to acquire precise anteroposterior view, aligning the lower endplates of the targeted lumbar vertebral body. Subsequently, to access the intervertebral foramen, the C-arm is tilted 20–30 degrees toward the direction of the proposed intervention. In the AR-NAVI group, upon activating the HMD, the physician evaluated the alignment between the 3D model of the reference marker in the AR view and the actual reference marker in the real world. After obtaining the first C-arm image in each group and confirming the reference point in the image, local anesthesia was applied to the site of needle insertion. Subsequently, the needle was advanced into the lower part of the pedicle of the target intervertebral foramen via the safety triangle approach. During the procedure, the AR-NAVI group utilized the aforementioned guidance provided by the augmented reality navigation system. In contrast, the control group relied on the appropriate guidance provided by the C-arm to insert the needle using the tunnel view technique. After confirming the final positioning of the needle, an administration of 3000 IU of hyaluronidase, diluted in 5 mL of 0.2% mepivacaine, was administered. In patients without diabetes, dexamethasone (5 mg) was mixed with the injection. Subsequent monitoring for potential side effects or adverse reactions was extended for 4 days after the procedure.

The primary outcome was the duration from the needle insertion until the final needle tip position. The secondary outcomes were the time to establish the needle insertion point, total procedure time, and number of radiographs taken. We established two time periods in which the assessment was performed. We defined the initial period (T1) as the time from immediately after obtaining the first C-arm image until the needle pierced the skin surface at the appropriate insertion point. The second period (T2) was defined as the duration from the needle insertion until the final needle tip position was confirmed to have reached the target using C-arm imaging. During the study period, the duration and number of radiographs taken in both groups were recorded. We evaluated complications within 30 min following the procedure. Compared to baseline, such as any increase in pain intensity measured on the numeric rating scale, and any changes in reflexes, sensory, or motor functions, were evaluated within one week of the procedure.

### 2.4. Statistical Analysis

In our pilot study using porcine models, the AR system group and control group required 155.6 ± 68.7 s and 250.8 ± 91.5 s, respectively, to place the needle at the target point after setting the insertion point. Based on the above results, the sample size for this study was calculated as 12 participants in each group (24 in total), with a two-sided significance level of 5% and a power of 80%. Considering an expected withdrawal rate of 10%, 14 participants were recruited for each group (28 in total).

The data were expressed as the mean ± standard deviation. Statistical analyses were performed using the SPSS statistical software (version 18.0; SPSS Inc., Chicago, IL, USA). The Mann–Whitney U test was used to compare the outcomes between the two groups. A *p*-value of <0.05 was considered significant.

## 3. Results

**Figure 3:**
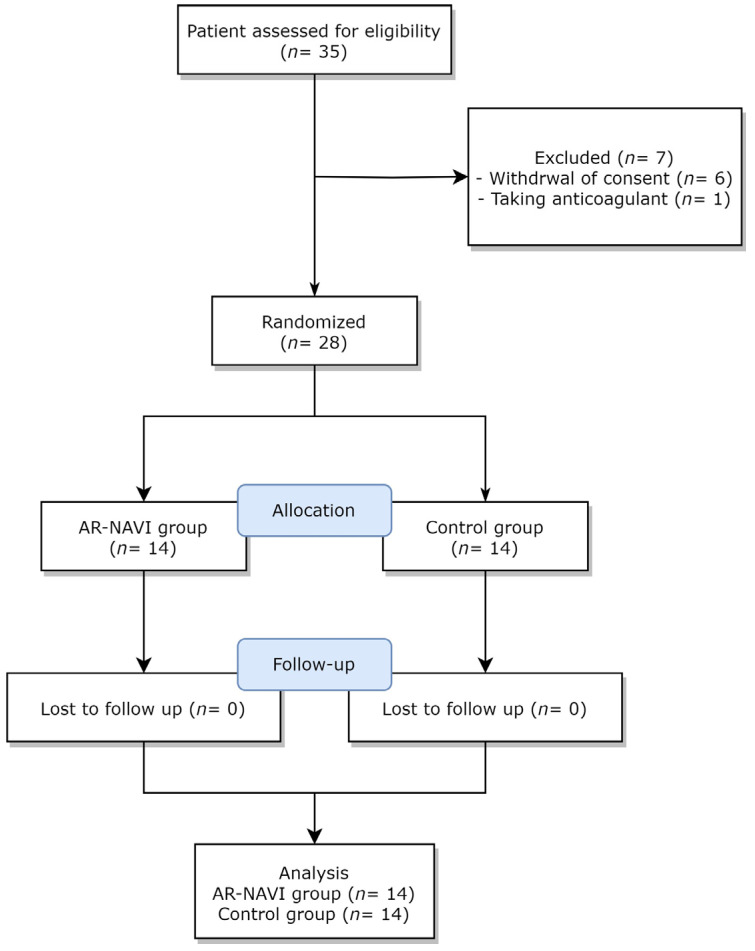
Flow diagram depicting the participants of the study. A total of 28 patients were randomly allocated into the AR-NAVI group and control group.

Among the initial 35 patients, 6 who withdrew their consent and 1 who was treated with anticoagulants were excluded (Figure 3). A total of 28 patients were randomly allocated to the AR-NAVI group and control groups, and all of them were included in the results analysis without any dropouts. The procedure was successfully performed in all patients within both groups, with no adverse effects. No significant differences were observed in the demographic or serological data of the participants in the two groups (Table 1). Similarly, pain scores and vital signs during the study remained comparable across both groups (Table 2). Within the initial 30 min and up to one week after the procedure, no adverse events occurred in both groups.

Regarding the time period between the acquisition of the first C-arm image and skin needle insertion, the AR-NAVI group demonstrated significantly shorter time requirements than the control group (28.29 ± 8.22 vs. 70.29 ± 27.33, *p* < 0.001). Moreover, the number of C-arm uses was lower in the AR-NAVI group than in the control group (1.29 ± 0.47 vs. 4.43 ± 1.34, *p* < 0.001).

The results of the assessments conducted during the time period between needle insertion to needle arrival at the target site and confirmation of the final needle position were similar. The AR-NAVI group exhibited significantly reduced duration within this period compared with that in the control group (58.57 ± 33.31 vs. 124.91 ± 41.14, *p* < 0.001). The required number of C-arms used during the period was also lower in the AR-NAVI group (3.79 ± 1.97 vs. 8.86 ± 3.94, *p* < 0.001) (Table 3). Total procedure time and total number of C-arm guides were also significantly reduced in the AR-NAVI group (86.86 ± 32.47 vs. 195.20 ± 53.89, *p* < 0.001; 5.07 ± 1.94 vs. 13.29 ± 4.36, *p* < 0.001).

## 4. Discussion

In this study, we performed transforaminal epidural blocks using AR-guided navigation on awake patients. To our knowledge, this is the first randomized controlled trial reporting the clinical application of an AR-assisted navigation system in interventional procedures other than surgery.

Various attempts have been made to apply AR-guided navigation in spinal surgery [8,11,22,23]. Most of these attempts have evidenced that the implant could be successfully inserted into the target site during surgery, demonstrating the usefulness and economic feasibility of AR-guided navigation in clinical practice [9,10,24].

However, unlike these studies on surgery, the present study had to overcome various challenges. First, performing the procedure in an outpatient setting, where patients cannot be immobilized under general anesthesia, necessitated real-time adjustments to account for patient anatomy movement during breathing or response to pain. We accomplished this by affixing markers to the patient’s back surface, continuously tracking it in real-time, and conveying its position relative to the reference marker through the HMD.

In addition, we tracked the movement of the small-diameter needle within the patient’s body. Although we traced it by placing a rigid-body marker at the handle of the needle, this did not reflect the exact location of the needle tip in the patient’s body. However, based on the results of this study, we have successfully validated the feasibility of conducting precise procedures utilizing our AR-guided navigation system in conjunction with periodic X-ray imaging acquired from a singular direction. Although this system could not provide information regarding the needle bending, the procedure could be successfully performed with limited X-ray imaging. Therefore, the information provided by our system about the insertion point and target orientation was sufficient for performing the procedure. However, the size of the tracking marker attached to the needle still caused discomfort during the procedure. By miniaturizing these markers or developing markerless tracking technology, it will be possible to offer a more convenient and comfortable guide.

Most navigation systems that utilize optical trackers encounter line-of-sight challenges [25,26]. This occurs when markers on the patient’s body or surgical tools are obscured by medical personnel or other equipment during the procedure. To address this issue, our system implements three optical trackers affixed at various positions. Although we successfully validated that this optical tracker configuration substantially mitigated the line-of-sight problem, a residual challenge persisted when the C-arm was positioned lower in the anterior-posterior direction.

Specifically, the extensive detector of the C-arm covered a significant portion of the patient’s dorsal surface, rendering the skin-attached markers invisible. To overcome this issue, we elevated the position of the C-arm, concurrently lowering the position of the optical tracker, and a deliberate space was created between the detector of the C-arm and the patient’s body. This adjustment effectively circumvented the line-of-sight obstruction, ensuring continuous visibility of the skin-attached markers even in scenarios involving an anterior–posterior C-arm configuration. Although there are still technical challenges to address, future efforts will necessitate addressing the line-of-sight distance limitation associated with these markers to facilitate continuous improvement and ultimately pave the way for the development of a markerless AR navigation system [27]. Such advancements are crucial for scaling and broadening the applicability of the AR navigation procedure.

Spine procedures are usually performed under the guidance of a two-dimensional projected image [1,2]. Although this can be overcome through extensive training, it can be difficult to perform if there is severe degeneration or deformity of the patient’s anatomy [28]. During the procedure, the needle must be held in the hand, placed in the proper position, and inserted in the correct direction, which is highly spatial. Our AR-guided navigation system projects the patient’s spinal structure three-dimensionally on the skin and intuitively presents the spatial relationship between instruments. Through this, a more intuitive and spatially accurate procedure using the human spatial recognition ability becomes possible which could not be utilized in the existing 2D image-guided procedure.

This study has some limitations. First, because all procedures were performed by a single clinician with more than ten years of experience, we could not verify whether doctors with different levels of experience could successfully perform the procedure using this system. As this was the first clinical trial related to this topic, only highly skilled physicians who were available at the time of the study performed the procedure. Second, because ethical constraints prevented precise measurement validation through CT scans while a needle is inserted into the patient’s body, the accuracy of the procedure could not be accurately evaluated. Third, it was difficult to account for alterations in spinal structure due to movement of the unanesthetized patient, which could lead to errors during the procedure. However, this study is considered significant as the insertion of a needle into the target intervertebral foramen and injection of drugs into the anterior epidural space were successfully performed in all patients, despite lacking a precise measurement. Fourth, a relatively small number of patients were included in the study. To enhance the generalizability of the results, it is essential to conduct further research involving a larger array of randomized controlled trials or multicenter studies. Furthermore, long-term follow-up was not performed to compare the treatment outcomes between the AR navigation and control groups. Further clinical studies are required to ascertain if there is a significant difference in the long-term treatment effects associated with the use of the AR navigation system. Despite these limitations, the results of this study will inform more reliable clinical trials and broader system applications.

## 5. Conclusions

Based on the outcomes of this randomized controlled study, our AR navigation system for pain procedures has demonstrated the potential to decrease both the duration of the procedure and radiation exposure during lumbosacral transforaminal epidural injection in an outpatient clinical setting. Nevertheless, further technological advancements and rigorous verification processes are essential to enhance the system’s efficacy and reliability.

## Figures and Tables

**Table 1:** Patients’ demographic characteristics and laboratory findings.

	AR-NAVI Group	Control Group	*p*-Value
Age (years)	55.36 ± 13.72	57.07 ± 14.98	0.755
Sex (Male/Female)	6 (42.9%)/8 (57.1%)	9 (64.3%)/5 (35.7%)	0.256
Height (cm)	164.66 ± 13.23	163.61 ± 8.35	0.804
Weight (kg)	69.71 ± 16.21	72.76 ± 11.39	0.570
WBC count (×109/L)	6.03 ± 1.07	7.13 ± 1.74	0.054
Platelet count (×109/L)	241.71 ± 78.60	260.71 ± 54.02	0.463
Glucose (mg/dL)	116.79 ± 44.24	118 ± 25.19	0.930
Blood urea nitrogen (mg/dL)	27.71 ± 41.58	26.55 ± 24.19	0.929
Creatinine (mg/dL)	0.82 ± 0.16	0.95 ± 0.21	0.095
aPTT (seconds)	31.06 ± 5.77	29.58 ± 7.75	0.575
PT/INR (seconds)	0.98 ± 0.05	0.97 ± 0.06	0.813
Side with radiating pain			0.246
Left	7	4	
Right	7	10	

Data are expressed as the number of participants (percentage) or mean ± standard deviation. WBC, white blood cells. aPTT, activated partial thromboplastin clotting time, PT/INR, prothrombin time test/international normalized ratio.

**Table 2:** Changes in vital signs and pain scores during the study period.

		AR-NAVI Group	Control Group	*p*-Value
Visit 2	Systolic blood pressure	126.64 ± 12.68	129.07 ± 10.00	0.578
	Diastolic blood pressure	81.93 ± 7.31	81.29 ± 8.56	0.832
	Heart rate	87.71 ± 15.42	82.00 ± 13.58	0.308
	Body temperature	36.59 ± 0.31	36.61 ± 0.20	0.775
	Numeric rating scale	5.71 ± 1.44	5.93 ± 1.54	0.707
Visit 3	Systolic blood pressure	126.64 ± 12.58	133.5 ± 10.49	0.129
	Diastolic blood pressure	85.36 ± 9.52	82.86 ± 7.96	0.458
	Heart rate	84.36 ± 14.22	83.21 ± 10.56	0.811
	Body temperature	36.59 ± 0.19	36.66 ± 0.18	0.276
	Numeric rating scale	4.50 ± 1.4	4.00 ± 1.88	0.432

Data are expressed as the mean ± standard deviation and number.

**Table 3:** Duration and number of C-arms used during the study period.

	AR-NAVI Group	Control Group	*p*-Value
T1			
Duration	28.29 ± 8.22	70.29 ± 27.33	*p* < 0.001 *
Number of C-arms guide	1.29 ± 0.47	4.43 ± 1.34	*p* < 0.001 *
T2			
Duration	58.57 ± 33.31	124.91 ± 41.14	*p* < 0.001 *
Number of C-arms guide	3.79 ± 1.97	8.86 ± 3.94	*p* < 0.001 *
T1 + T2			
Duration	86.86 ± 32.47	195.20 ± 53.89	*p* < 0.001 *
Number of C-arms guide	5.07 ± 1.94	13.29 ± 4.36	*p* < 0.001 *

Data are expressed as the mean ± standard deviation. T1: time from immediately after obtaining the first C-arm image until the needle pierced the skin surface at the appropriate insertion point; T2: duration from needle insertion until final needle tip position was confirmed to have reached the target using C-arm imaging. * Indicates statistically significant between-group differences (*p* < 0.001).

## Data Availability

The datasets used and/or analyzed during the current study are available from the corresponding author on reasonable request.
